# Public Health Coordinator – How to Promote Focus on Social Inequality at a Local Level, and How Should It Be Included in Public Health Policies? Comment on "Health Promotion at Local Level in Norway: The Use of Public Health Coordinators and Health Overviews to Promote Fair Distribution Among Social Groups"

**DOI:** 10.15171/ijhpm.2018.74

**Published:** 2018-08-25

**Authors:** Wenche Bekken

**Affiliations:** Department of Social Work, Child Welfare and Social Policy, Oslo Metropolitan University, Oslo, Norway.

**Keywords:** Inequality in Health, Universalism, Targeting, Public Health Coordinator, Norway

## Abstract

The Norwegian Public Health Act of 2012 (PHA)^1^ states that the social causes of inequality in health have not been devoted sufficient attention in Norwegian health policy. Different means have been implemented to pay more attention to health inequalities at a local level, one is the use of a designated public health coordinator (PHC). Hagen et al^2^ reveals in a new study, however, that the presence of PHCs’ does not add to the priority of reducing inequality as a health objective. This negative association is, by the authors, explained by a widespread use of coordinators before the Act, and as such, not really a new measure. Another factor emphasized is that the PHC position is not empowered by bureaucratic backing. I agree with these explanations. However, the study by Hagen et al2 lacks a critical discussion of how the role of the PHC is situated in an administrative intersection between national health policy based on universal initiatives and social policy in the municipalities historically driven by a focus on poverty and specific target groups. This commentary reflects upon how social inequalities in health at a local level and the responsibilities imposed on the municipalities contest the principals of universalism. The tension between universalism and selectivity needs to be more prominent in the debate on how health inequalities should be abated at the local level, if universalism shall prevail as the overarching principle in Norwegian health policies. The commentary concludes by asking for a more nuanced discussion on how work with health related social problems can support universalistic initiatives. It is also suggested as a task for the PHC to make sure that public health initiatives are systematically evaluated. Documentation of effects will provide knowledge needed about how initiatives affects the social gradient over time.

A newly published article Hagen et al^2^ reveals results from a study of Norwegian municipalities on how Health in all Policies (HiAP) tools, overviews and health coordinators, affects the prioritizing of the goal of fair distribution among social groups at the local level. The study analyses the situation before and after implementation of the 2012 Public Health Act (PHA).^2^ The health promotion perspective in the PHA is broadly defined. It is ‘health in all we do’ and central principals are equalizing social and living conditions, user involvement and sustainable development. The PHA also underscores that public health policy shall focus on social inequality in health, that municipalities are responsible for planning and implementation of initiatives, and that health for all shall be achieved by universal measures. However, it is also underscored that targeting specific problem groups within universal programs is necessary if for example mental health problems, violence, and substance misuse are to be reduced (p. 12).^1^ In local public health policies, health overviews and a specific position as public health coordinator (PHC) are used to monitor the population’s health and to organize public health policy. These tools aims at enhancing attention to social inequality as well as on the conditions for preventing or reducing bad health.

Hagen et al^2^ conclude that after the implementation of the PHA, development of public health overviews have increased the leverage given to fair distribution. On the other hand, the presence of PHCs’ does not add to the priority of equality. The negative association is explained by a practice in municipalities to use staff to coordinate public health work even before the implementations of the Act. Other factors emphasized are that the PHC position is not empowered by bureaucratic backing, and often unskilled part time working personnel hold the role, often as little as one day a week. The finding that PHCs’ does not enhance focus on social inequality is interesting, but in afterthought not surprising. Some insights can be mentioned. There is still a need to emphasize the effect of social conditions on health^2,3^ and there are several discussions on obstacles related to solving health inequality problems.^3-8^ Political attention to social inequality in public health is fairly recent, and the PHCs’ have their main focus on planning and coordination of the local health policy promoting health in all policies.^2^ Public health activities at the local level are predominantly initiatives as recreation, area planning, nutrition, and cultural activities.^9-11^ These are initiatives for all, and social inequality objectives are rarely explicitly emphasized. Any effect the PHC may have on increasing the focus on social inequality, can first be assessed when it is clearly defined how the coordinator shall emphasize social inequality. The coordinator should be given priority and status, and Hagen et al^7^ as well as others^3,8^ underscore an important agency with executive power. The impact of the PHC should be evaluated after the PHC has achieved this.

In the PHA, social inequalities are exemplified by problem areas as violence, mental health problems, and substance abuse. In order to reduce social inequalities it is necessary to employ a combination of universal and targeted measures (p. 12).^1^ It is documented that such problems are still managed at sectorial level even though collaboration across sectors is favoured (p. 23).^3^ This implies that a health perspective easily overshadows inequalities in social living conditions. A social perspective should be emphasized more clearly in cross-sectorial collaboration, if ‘health in all policies’ is to be effective. To reduce social inequalities in health, inequalities in living conditions must be the first premise to work with and to argue for, and conceive health as a result of this. I believe that PHCs’, in their work to stimulate cross-sectorial activities, are more concerned with arguing for health and why collaboration is needed to promote health in all we do, such as planning for recreational facilities and public accessible areas. The PHCs’ role in how to emphasize the social aspect in their work, must be more clearly stated. It would also be relevant to ask PHCs’ what they experience as obstacles in their work with social inequality and to document how PHCs’ reflect upon the relationship between health and social conditions. I do believe that opinions differ among the PHCs’ in the 428 Norwegian municipalities, and research is needed to verify the proposition.

It really is a paradox that it is necessary to emphasize social inequality in health when public health in itself is about implementing initiatives to reduce social health differences in living and health conditions. A fundamental objective of the Norwegian welfare state has been to improve the population’s health.^1,12^ The social determinants of health have however not been adequately emphasized. In Norway, the post war welfare policy had an equalizing effect. Nevertheless, class differences in health has in fact increased.^13^

This leads to the question of how to reconcile politics to address social health inequalities at a local level with the principle of universal measures at a national level. I believe that social inequalities in health basically should be addressed within universal policies. However, this principle must not be at the expense of a focus on what individuals or sub groups actually need of support to be able to take good choices and sustain a good life. Examples are refugees, children living in poverty, families with need for social care services. What their needs and attitudes are toward receiving welfare, can help us to design effective universal initiatives. This argument should not be confused with the neo-liberal argument based on normative, moral considerations of individuals’ responsibility to solve their own problems.

In the tradition of social work, a key question is how to help people by universal measures and targeted initiatives. There is a continuous evaluation of best practice and how to work with groups and individuals without stigmatizing them.^14^ The framing of a problem often involves identifying patterns related to individuals’ and groups’ behaviours and attitudes. It is crucial not to present information about groups that can foster stereotyped and condescending attitudes. On the other hand, transparency about social problems may prevent misconceptions. User involvement and initiatives adjusted to local conditions, are necessary in social work,^15,16^ which also is emphasized in the PHA.^1^ A focus on social inequality in health means to pay attention to those individuals who do not take advantage of public health campaigns and universal initiatives. Therefore, it is necessary to clarify how targeted work might be needed in order to achieve the intended effect of universal initiatives. At the same time, it is necessary to discuss what targeting really means in a concrete local context. Are for example employment programs a universal initiative or is it targeting within universalism? Are additional conversations groups to persons enrolled in employment programs or activities enhancing coping skills, regarded as targeted or not? I leave these questions open, and will argue that we need discussions on how specific health problems are related to social conditions. I also believe, that it can be interesting to ask PHCs’ if they are familiar with the concepts of universalism and targeting within universalism.

Knowledge about how health is distributed among inhabitants of Norwegian municipalities, gives insights into local health challenges. The problem spectre in a rural agricultural municipality differs significantly from that of an urban area. Also, municipalities within a region with statistically similar challenges, most likely, due to different cultures and history, have to address these within different initiatives.

At present, there are not many thoroughly evaluated public health initiatives targeted at socially determined patterns of health inequality.^16^ There is need for more knowledge both on what and which initiatives have effect on reducing inequality, and in the long run, have the desired impact on the social gradient in the patterns of the population health as a whole. Thus, municipalities should increase collaboration with research institutions.

The PHC role must be empowered by bureaucratic backing, as Hagen et al suggests.^2^ An important task for the PHC can be to ensure that local public health initiatives are evaluated. It should be asked if ‘health in all policies’ favours the health perspective more than how social conditions actually shape good health and quality of life? If local politicians and administrators are to have greater responsibility for implementing public health initiatives, as well as to focus more systematically on fair distribution, it should be led by a policy of universal initiatives. However, the discourse must to a greater extent underscore how PHCs’ are to work with the complexity of poverty, what social living conditions consists of, and how quality of life is distributed locally, in order to support initiatives for all. If the role of the PHC is strengthened and the social inequality focus is more solidly established as an integral part of the position, the PHC role can be evaluated as useful or not. Further research should investigate what PHCs’ conceive of the concepts of universalism and targeting within universalism. It should also be investigated if and how PHCs’ promote social living in public health work. I will strongly propose a qualitative in depth study.

## Ethical issues

Not applicable.

## Competing interests

Author declares that she has no competing interests.

## Author’s contribution

WB is the single author of the paper.
